# Posttraumatic growth, depressive symptoms, posttraumatic stress symptoms, post-migration stressors and quality of life in multi-traumatized psychiatric outpatients with a refugee background in Norway

**DOI:** 10.1186/1477-7525-10-84

**Published:** 2012-07-23

**Authors:** Dinu-Stefan Teodorescu, Johan Siqveland, Trond Heir, Edvard Hauff, Tore Wentzel-Larsen, Lars Lien

**Affiliations:** 1 Innlandet Hospital Trust, PO Box 104, Brumunddal, N-2381, Norway; 2 R & D Department, Mental Health Sevices, Akershus University Hospital, Lørenskog, Norway; 3 Norwegian Centre for Violence and Traumatic Stress Studies, Kirkeveien 166, Oslo, N-0407, Norway; 4 Division of Mental Health and Addiction, Oslo University Hospital, Kirkeveien 166, Oslo, N-0407, Norway; 5 Institute of Clinical Medicine, Faculty of Medicine, University of Oslo, Oslo, Norway; 6 Centre for Child and Adolescent Mental Health, Eastern and Southern Norway, Postboks 4623 Nydalen, Oslo, N-0405, Norway; 7 Biostatistics and Epidemiology Unit, Oslo University Hospital, Postboks 4956 Nydalen, Oslo, N-0424, Norway

## Abstract

**Background:**

Psychiatric outpatients with a refugee background have often been exposed to a variety of potentially traumatizing events, with numerous negative consequences for their mental health and quality of life. However, some patients also report positive personal changes, posttraumatic growth, related to these potentially traumatic events. This study describes posttraumatic growth, posttraumatic stress symptoms, depressive symptoms, post-migration stressors, and their association with quality of life in an outpatient psychiatric population with a refugee background in Norway.

**Methods:**

Fifty five psychiatric outpatients with a refugee background participated in a cross-sectional study using clinical interviews to measure psychopathology (SCID-PTSD, MINI), and four self-report instruments measuring posttraumatic growth, posttraumatic stress symptoms, depressive symptoms, and quality of life (PTGI-SF, IES-R, HSCL-25-depression scale, and WHOQOL-Bref) as well as measures of social integration, social network and employment status.

**Results:**

All patients reported some degree of posttraumatic growth, while only 31% reported greater amounts of growth. Eighty percent of the patients had posttraumatic stress symptoms above the cut-off point, and 93% reported clinical levels of depressive symptoms. Quality of life in the four domains of the WHOQOL-Bref levels were low, well below the threshold for the’life satisfaction’ standard proposed by Cummins.

A hierarchic regression model including depressive symptoms, posttraumatic stress symptoms, posttraumatic growth, and unemployment explained 56% of the total variance found in the psychological health domain of the WHOQOL-Bref scale. Posttraumatic growth made the strongest contribution to the model, greater than posttraumatic stress symptoms or depressive symptoms. Post-migration stressors like unemployment, weak social network and poor social integration were moderately negatively correlated with posttraumatic growth and quality of life, and positively correlated with psychopathological symptoms. Sixty percent of the outpatients were unemployed.

**Conclusions:**

Multi-traumatized refugees in outpatient clinics reported both symptoms of psychopathology and posttraumatic growth after exposure to multiple traumatic events. Symptoms of psychopathology were negatively related to the quality of life, and positively related to post-migration stressors such as unemployment, weak social network and poor social integration. Posttraumatic growth was positively associated with quality of life, and negatively associated with post-migration stressors. Hierarchical regression modeling showed that posttraumatic growth explained more of the variance in quality of life than did posttraumatic stress symptoms, depressive symptoms or unemployment. It may therefore be necessary to address both positive changes and psychopathological symptoms when assessing and treating multi-traumatized outpatients with a refugee background.

## Introduction

Many refugees in Norway have been exposed to multiple traumatic events in their home countries, such as imprisonment in concentration camps, torture or multiple rapes [1]. As a consequence of this they can suffer from a variety of psychiatric problems for many years after resettlement. In spite of exposure to such overwhelming traumatic events, some refugees also report positive personal changes related to these highly negative events, often referred to as posttraumatic growth.

This phenomenon of benefiting or growing psychologically from traumatic events has been long recognized throughout human history, and in the 19^th^ century Nietzsche famously said “That which does not kill us makes us stronger” [2], indicating the potential for personal growth after a traumatic event. After the Second World War, Victor Frankl began the modern inquiry of perceived positive changes [3] and later several researchers have empirically identified the same phenomenon, some calling it “benefit finding” [4], “stress-related growth” [5], “adversial growth” [6], and “posttraumatic growth” [7]. Tedeschi and Calhoun defined posttraumatic growth (PTG) as a “positive psychological change experienced as a result of the struggle with highly challenging life circumstances” ([8] page1). According to Tedeschi and Calhoun, positive changes can be divided into five sub-domains: relating to others, new possibilities, personal strength, spiritual change and appreciation of life. There is a growing volume of research providing evidence for psychological growth in a variety of populations (children, adolescents, and adults) exposed to diverse traumatic events. Posttraumatic growth has been identified in refugees and immigrants [9-13], adult civilians exposed to war [14,15], civilians exposed to terrorism [16-18],veterans of war [19,20], former child soldiers [21], former war prisoners [22-25], former political prisoners [26], and in victims of interpersonal violence [27-30]. For PTG to be more than an interesting concept for academic psychology, its relation to health, both physical and mental, and quality of life are essential, and research thus far has been inconclusive about these relationships [31-33].

### Posttraumatic growth and posttraumatic stress symptoms

PTG is related to the dramatic impact of a traumatic event, which is followed by symptoms of psychological distress and PTSD symptoms. It is not essential to the development of PTG that the person experiences a full diagnosis of PTSD, but some psychological distress is essential [8]. Tedeschi and Calhoun proposed that posttraumatic growth and posttraumatic stress may be largely two different dimensions, thus, it is possible to report both growth experiences and distress in trauma survivors [8,34]. There are conflicting results from empirical studies on posttraumatic growth theory. Many studies report a positive relationship between PTG and posttraumatic stress symptoms in refugees [11], people exposed to community violence [28], military combat [19], acts of terrorism [17,35,36], war imprisonment [37] and other trauma [38-41] . However, several studies have found a negative relationship between PTG and PTSD [15,16,18,42-44] while one study reported that PTG and PTSD were unrelated [45].

Other researchers have suggested a curvilinear relationship between PTG and PTSD, where higher scores of PTG are associated with mild PTSD symptoms, and lower PTG scores are associated with high and low PTSD symptoms [30,46,47]. The conflicting findings on the relationship between PTG and PTSD might be explained by mediating factors unaccounted for in previous research. One such mediating factor is time elapsed since the traumatic event [7,31,48]. In respone to this, Zoellner and Maercker proposed a two faced model of PTG [49], the Janus faced model. This model suggests that PTG has one constructive, self-transcending side and another self-deceptive, illusory side. The first type of change is thought to be related to very longterm changes whereas the latter is related to shortterm compensatory coping mechanisms. This theory has received some empirical support. A majority of studies on PTG and PTSD measured shortly after a traumatic event report a positive correlation between PTG and PTSD [17,19]. With the passing of time this correlation attenuates or becomes negative as a more substantial and authentic form of PTG develops. The action focused theory of growth [16] underscores the necessity for action rather than pure cognitions in order to experience a thorough transformation and a genuine and lasting growth. Thus, the Janus faced model and the action focused theory share some predictions regarding the relationship between PTG and PTSD, namely, that the positive relationship between PTG and PTSD observed in the first period after the traumatizing event should attenuate over time, possibly because the passage of time presents chances to set new ideas and perspectives into action.

### Quality of life, growth and psychopathology

Improved quality of life is a major outcome in health care, and therefore it is important to assess its relationship to both positive changes (PTG) and symptoms of psychopathology (posttraumatic stress or depressive symptoms). Yet very few studies of refugees resettled in a Western country have assessed the relationship between posttraumatic growth, posttraumatic stress, depressive symptoms and quality of life.

The findings regarding the relationship between PTG and quality of life have been mixed.

Some studies have reported a positive association between quality of life and PTG among cancer patients [50-53], and traumatized students [54], whereas other studies have reported no relationship [55-58]. A meta analytic review investigating the relationship between PTG and quality of life found that the two constructs were unrelated, mainly due to the use of global measures for quality of life that included both mental and physical health [31].

Previous research seems more consistent regarding the relationship between psychopathology and quality of life. Generally, studies have reported a negative relationship between PTSD or depression and quality of life in veterans [59], cancer survivors [60], refugees with somatic pain [61], war-wounded refugees [62], and tortured refugees resettled in Denmark [63-65].

### Quality of life, growth, psychopathology and post-migration stress

Refugees are exposed to both pre- and post-migration stressors, and together these factors have a direct influence on quality of life [62,66]. Pre -migration variables such as the nature and severity of the trauma or demographic characteristics still influence a person’s quality of life in the resettlement country, but it seems that post-migration stressors like acculturation stress, weak social support, loss of social roles, unemployment, and cultural bereavement have an even greater negative impact on health, especially from a long-term perspective [67].

One post-migration stressor is the loss of one’s social network from the home country, causing a weak social network which reduces the person’s coping capacity for traumatic exposures, including the acculturation process itself [68]. Several studies have found a positive association between PTG and social support in refugees [10], and in former prisoners of war [25], while one study found no association [69].

Further, social support and social network have been found to have a negative association with PTSD and depression in resettled refugees [70,71].

Quality of life has been found to be positively associated with social support, and negatively associated with mental health symptoms among refugees resettled in Sweden [66].

Employment is one of the most important markers of achievement for the majority of refugees, especially in males, due to the fact that employment is closely related to a sense of identity as well as to a feeling of self-worth, which are under constant threat during the migration and resettlement process [72,73]. Further, employment is seen by many refugees as a means to financial independence, and closely related to a good quality of life and mental health status in the country of resettlement [74-76]. Unemployment is acknowledged by many refugees as a major source of post-migration stress which has profound negative implications for both health (physical and mental) and quality of life [77-81]. Unemployment has been identified as a risk factor for the development of mental health problems in immigrants and refugees [70,82-87].

Quality of life has been found to be positively associated with employment among tortured refugees resettled in Denmark [64], while posttraumatic growth was also positively associated with paid work in people with traumatic brain injury (TBI) [88].

Social integration in the country of resettlement can be stressful, and PTSD can further hinder language acquisition [89], cause social isolation [90] and increase acculturation stress [91]. A positive association was found between mental health symptoms and acculturation stress in both immigrants and refugees [92-94]. Poor acculturation was found to be positively related to poor self-reported health among immigrants from Poland, Turkey and Iran resettled in Sweden [95].

### The present study

Few studies have investigated quality of life in relation to posttraumatic stress symptoms, depressive symptoms and PTG among refugee populations. Some of these studies have used self-made abbreviated versions of questionnaires, thus raising the question of the validity of the results. Furthermore, few studies have included psychiatric outpatients with exposure to war and war related situations, and further research on this population is needed. The majority of PTG research has investigated only the positive changes that occur after exposure to a traumatic event. Action focused growth theory, however, also suggests that “PTG that occurs in the context of taking action is likely to relate to lower levels of psychological distress “[42]. We therefore also sought to investigate negative psychological symptoms and their relationship to posttraumatic growth and quality of life. Based on the literature, we investigated the following aims:

### AIMS

1. To assess the presence and level of posttraumatic growth, posttraumatic stress symptoms, depressive symptoms, post-migration stressors and quality of life, and their relationship in a population of multi-traumatized refugees years after resettlement in Norway.

2. To test a model for predicting quality of life among multi-traumatized refugees.

We proposed the following hypotheses:

1. Posttraumatic growth will be negatively associated with psychopathology and positively associated with quality of life.

2. Posttraumatic stress and depressive symptoms will be negatively associated with quality of life and positively associated with post-migration stressors such as weak social network, poor social integration and unemployment.

3. Post-migration stressors like weak social network, poor social integration and unemployment will be negatively associated with quality of life and posttraumatic growth.

## Method

### Participants

Sixty-three patients were recruited for the study from the outpatient departments of four hospitals from South-Eastern Norway between November 1^st^, 2008 and November 1^st^, 2009. Two subjects rescinded consent, so that sixty-one patients were retained in the study. Six patients failed to return the questionnaires or to complete several scales and were thus excluded. Fifty-five persons were included in the final sample, where 32 were men (58%) and 23 (42%) were women with an age range from 21 to 61 years.

The patients were referred to outpatient treatment by their General Practitioners for a variety of psychological and psychiatric problems. For the majority of the patients the problems were related to pre-migration exposure to traumatic events, as well as experiencing post-migration acculturation difficulties. Any patient with a refugee background receiving outpatient treatment in any of these four hospitals who had been exposed to a traumatic event according to the criteria of DSM-IV-TR was asked to participate in the study. Further inclusion criteria included being between the ages of 18 and 61 years old, having a permanent residence in Norway and being sufficiently proficient in spoken and written Norwegian. Patients were excluded if they suffered from any serious medical or neurological illness or organic mental disorder, were strongly sedated under current medication, had an active psychotic episode, were currently considered a high suicide risk or had insufficient knowledge of the Norwegian language. We were not able to determine the total number of patients with a refugee background receiving treatment during the study period among the outpatient departments from the four hospitals due to internal hospital reporting regulations that do not require the ethnicity of patients.

#### Procedures and ethics

Instruments included two structured clinical interviews, and a series of self-report questionnaires completed at home and returned to the interviewing researcher, a clinical psychologist and first author (D-S. T.) by post within a week. The duration of the clinical interview varied depending on the patient’s psychological condition and the personal history of exposure to multiple traumas. The average clinical interview time was two hours, excluding pauses which were taken at regular intervals, and at the patient’s request.

Written informed consent was collected from all the patients prior to the clinical interview. At the completion of the interview patients received 200 NOK (EUR 25) in cash as well as reimbursement covering travel expenses. The patients also received stamped envelopes along with the self-reported questionnaires to be completed and posted within a week. One telephone follow-up reminder was made if the questionnaire was not returned after one week. We did not inquire as to whether or not patients received language help when completing the questionnaires at home.

The study was conducted in accordance with the Helsinki Declaration for research involving human subjects, and was approved by the Committee for Medical Health Ethics - Region East of Norway (REK-East).

#### Measures

The Life Events Checklist (LEC) is a 17-item screening instrument for potentially traumatic events to be administered before the Clinician Administered PTSD Scale (CAPS), which is intended for assessing the presence of a PTSD diagnosis. LEC does not have a specific timeframe, specific for exposure to potentially traumatic events is the whole lifespan. Other studies have found the LEC to have acceptable reliability [96,97]. The LEC uses five answer categories, ranging from “It happened to me” to “Not relevant”. In order to assess the strongest impact of a potentially traumatic event we selected yes/no responses from the first answer category “It happened to me”. We used a Norwegian translation of the LEC used in several research projects by the Norwegian National Centre for Violence and Traumatic Stress.

The Structural Clinical Interview for DSM-IV- TR PTSD Module (SCID-PTSD) is a clinical structured interview for the diagnosis of PTSD [98,99]. Each patient was asked about his or her worst traumatic experience ever and each symptom from the clinical interview in regards to this traumatic experience was assessed. The SCID-PTSD contains 6 criteria which need to be fulfilled in order for a current PTSD diagnosis to be given, which is in agreement with the DSM-IV-TR diagnostic manual [100]. We used a Norwegian translation developed in July 1996 by the Centre for psychological clinic research, Institute of psychology which has been widely used.

The MINI International Neuropsychiatric Interview 5.0.0 (MINI) is a structured diagnostic interview that assesses 25 axis DSM-IV disorders, one axis II disorder (anti-social personality disorder) and suicidal risk [101]. We excluded from our clinical interview protocol the PTSD module and the anti-personality disorder module.

The MINI English version has shown good psychometric properties and is currently used in more than 100 countries according to Medical Outcomes Systems Inc. We used a Norwegian translation developed by Kari Ann Leiknes and colleagues which was validated among a sample of inpatients from an acute psychiatric ward in Norway [102].

The Impact of Event Scale-Revised (IES-R) [103] is a 22-item instrument to measure the severity of post-traumatic stress symptoms during the last week. The items are rated on a 5-point Likert scale from 0 (not at all) to 4 (extreme), with total scores ranging from 0 to 88 and where higher scores indicate a greater symptom load. The original version in English showed good internal consistency with Cronbach’s alpha coefficients ranging from 0.81 to 0.91 [104]. We used a Norwegian translation of the IES-R that in a previous study produced the following Cronbach’s alpha coefficients: total scale = 0.94, Intrusion subscale = 0.89, Avoidance subscale = 0.84 and Hyperarousal subscale = 0.88 [105]. In our study we calculated the following Cronbach’s alpha coefficients for the IES-R: total scale = 0.94, Intrusion subscale = 0.91, Avoidance subscale = 0.79, and Hyperarousal subscale = 0.85. We used a cut-off score of ≥ 33 to indicate a PTSD diagnosis [106].

The Hopkins Symptom Check List-Depression scale (HSCL-25-Depression) is a self-report scale with 15 items assessing depressive symptomatology during the last week. The items are rated on a 4-point Likert scale from 1 (not at all) to 4 (extremely), with total scores ranging from 15 to 60 and where higher scores indicate a greater symptom load. The original scale’s Cronbach’s alpha coefficient was = 0.85. [107]. Validated versions used in three refugee populations showed sensitivity and specificity for the presence of depression of 0.88 and 0.73, respectively [108]. The HSCL-25 has been translated into Norwegian and validated among immigrant populations in Norway [109,110]. We used a cut-off score of > 1.75 to indicate clinical symptoms of depression. In our study we calculated a Cronbach’s alpha coefficient = 0.92.

The Posttraumatic Growth Inventory Short Form (PTGI-SF) is a 10-item self-report scale [111] derived from the 21-item PTGI scale [7]. It contains five factors (2-items in each): relating to others, new possibilities, personal strength, spiritual change and appreciation of life. The items are rated on a 6-point Likert scale from 0 (no change) to 5 (very great degree of change), with domains scores ranging from 0 to 10 and where higher scores indicate a greater positive change. The original Cronbach’s alpha coefficient for the total PTGI-SF scale was α = 0.89 [111]. The Norwegian version of the PTGI used in this study has previously showed good psychometric properties, with a Cronbach’s alpha for the full scale of 0.86 [41]. In our study we calculated a Cronbach’s alpha coefficient for the full scale = 0.89.

The World Health Organization Quality of Life-Bref scale (WHOQOL-Bref) is a self-report, abbreviated version of the WHOQOL-100 containing 26 items divided into four domains and 2 general items. The first general item measures overall quality of life, the second general item measures overall general health, while the first domain measures physical health (7 items), the second domain measures psychological health (6 items), the third domain measures social relationships (3 items), and the fourth domain measures environmental quality of life (8 items). The WHOQOL-Bref was originally developed for use as a cross-cultural instrument and validated across 15 field centers worldwide [112]. The items are rated on a Likert scale from 1 to 5, with domains scores ranging from 4 to 20 where higher scores indicate a better quality of life. The original version of the WHOQOL-Bref produced the following Cronbach’s alpha coefficients: physical health = 0.80, psychological health = 0.76, social relationships = 0.66 and environment = 0.80 [113]. Studies using the Norwegian version used in the present study have previously reported the following Cronbach’s alpha coefficients for the four domains: physical health = 0.84, psychological health = 0.82, social relationships= 0.60 and environment = 0.79 [114]. In our study we calculated the following Cronbach’s alpha coefficients: physical health =0.77, psychological health = 0.77, social relationships = 0.54 and environment = 0.78, and the total WHOQOL-Bref 26 items = 0.92.

Social network was measured using one question: “How many good friends do you have?” (“Count those whom you can talk to in confidence and who can help you when you need help”) [115]. A higher number of friends indicated a better social network. The present variable was previously used in a community survey in Norway as a part of a self-report questionnaire [116]. The variable was reversed in order to measure weak social network.

Social integration was used in order to assess the level of integration into Norwegian society, and was measured by an index based on four items: (1) Knowledge of the Norwegian language; (2) Reading Norwegian newspapers last year; (3) Being visited by Norwegians last year; (4) Help/support from Norwegians last year. An index score was calculated (range 4 to 16), by adding the scores for the four items, with higher scores indicating higher integration. The final score of the resulting variable was reversed in order to measure poor social integration into Norwegian society. The index used in the present study has been previously used in another Norwegian study on social integration and mental health among immigrants in Norway [117]. In our study we calculated a Cronbach’s alpha coefficient of 0.72.

Employment was assessed by the question “Are you in paid employment?” with three response alternatives: (0) “Not in paid employment”; (1) “Part-time paid employment”; (2) “In full-time paid employment”. The response alternatives (1) and (2) were collapsed into a single category “In paid employment” thus creating a dichotomous employment variable. The resulting variable was reversed in order to measure unemployment.

Religion was assessed with one question” To which religion do you belong?” with 6 response alternatives: (1) Christianity; (2) Islam; (3) Hinduism; (4) Buddhism; (5) Other religion, and (6)”I don’t belong to any religion”. The answer categories 3, 4 and 5 were collapsed into one category with the label ”other religion”.

### Statistical analyses

Descriptive analyses included frequencies and means of demographic variables. For key variables we reported means, standard deviations and range. We conducted bivariate analyses to assess relationships between variables, using Spearman’s correlations for normally distributed continuous variables and Kendall’s tau b for categorical variables.

Histograms and scatter diagrams were used to assess the normality of the distribution, linearity and homoscedasticity for all continuous variables.

Multiple linear regression analyses were used to assess whether the independent variables (gender, scores of HSCL-25-D, IES-R, PTGI-SF, and unemployment) had significant associations with the dependent variables (WHOQOL-Bref four domains). The predictors chosen in the analyses were included on the basis of previous scientific findings. The results were not corrected for multiple comparisons because of the exploratory nature of this study. Hierarchical regressions were also used to assess the individual contribution of each predictor variable (scores of HSCL-25-D, IES-R, PTGI-SF, and unemployment) to the total variance on the dependent variables of the four quality of life domains. Preliminary analyses indicated no violations from the principles of normality, linearity, multicollinearity and homoscedasticity. Multicollinearity was examined using the variance inflation factor (VIF). Homoscedasticity was examined through a visual inspection of the scatterplots of residuals by predicted values. We found no multivariate outliers using Mahalanobis distances with an α set at 0.001. [118]. Missing data was handled by using only complete cases in the multivariate analyses. The missing values for the WHOQOL-Bref were handled according to instructions from the WHOQOL-Bref manual [119].

Cronbach’s alpha coefficients were calculated for all scales. All statistical tests were two-tailed with an alpha level set at 0.05 and were performed using IBM SPSS Statistics 19 software package (IBM SPSS Statistics Inc. Armonk, New York, USA). Power analyses for the PTGI coefficient in the regression analyses were based on simulations in the estimated models with actual data for the independent variables using the R Statistical Environment (The R Foundation for Statistical Computing, Vienna, Austria).

## Results

### Demographic and pre-migration characteristics

The mean age for men was 44.0 (SD=10.4) years, the mean age for women was 39.3 (SD=6.2) years, and the majority were Muslims (64%). The mean length of time of living in Norway was 16.7 years (SD = 7.1), and the mean time since the traumatic exposure was 17.7 years (SD= 9.4).

Forty-three (79.6%) patients were exposed to a war zone situation, and 22 (36.1%) were exposed for more than 3 years. The mean number of total types of potentially traumatic events as measured by LEC ranged from 2 to 15 types (Mean= 9.8, SD= 2.7). The patients were suffering from a variety of psychiatric disorders, with a majority (94.5%) suffering from anxiety disorders and affective disorders (80%).

### Posttraumatic growth, posttraumatic stress symptoms, depressive symptoms, and quality of life

The mean PTGI-SF total score was 22.6 (SD = 10.1), and is equivalent to 47.4 on the PTGI 21-item questionnaire. The mean for the item response on a 5-point scale was M = 2.34, SD = 1.01, corresponding to an amount of growth between”small and medium”. Even if all the patients reported some amount of growth, with 30.9% reporting”a very great degree”, the total amount of growth was low. The factors with the highest scores were: appreciation of life (M = 2.65, SD = 1.15), spiritual change (M = 2.25, SD = 1.34), and personal strength (M = 2.23, SD = 1.33). The factors with the lowest scores were: new possibilities (M = 2.18, SD = 1.27) and relating to others (M = 2.20, SD = 1.34). The most rated individual items were: “I am able to do better things with my life” (64.1%), “I established a new path for my life” (57.7%), and “I changed my priorities about what is important in life”(57.1%).

The IES-R total means score was 51.7 (SD = 17.1), and 80% of the outpatients scored above the cut-off point. The HSCL-25 depression subscale had a mean score of 2.7 (SD = 0.64), and 93% of the outpatients scored above the cut-off point.

The quality of life results from the WHOQOL-Bref scale show that 10.9% of the outpatients were satisfied with their general health, and 7.4% said that they had a good quality of life.

The WHOQOL-Bref domains had the following means and standard deviations: physical health (M = 9.2, SD = 2.4), psychological (M = 9.5, SD = 2.5), social (M = 10.9, SD = 3.1) and environmental (M = 10.7, SD = 2.4).

In order to evaluate the WHOQOL-Bref levels based on a gold standard for subjective well-being as proposed by Cummins [120], we transformed the domain scores (4–20) to the full scale domain scores for WHOQOL-100 (0–100). Cummins proposes that”a population standard for’life satisfaction’ can be expressed as 75.0 ± 2.5 percent of the measurement scale maximum score” [120]. The new domain means corresponding to the total scale were: physical health (M = 32.5; SD = 14.7), psychological health (M = 34.6; SD = 15.5), social relationships (M = 43.4; SD = 19.7), and environmental (M = 43.1; SD = 15.1). All levels of the quality of life domains were well below the threshold for the’life satisfaction’ standard proposed by Cummins.

### Post-migration stress factors

The average number of good friends was 3.0 (ranging from 0 to 11) and a large proportion of patients reported having no friends at all (25.9%). Scores below the mean for social integration were found in 45.5% of patients, and unemployment rates were very high at 59.6% (see Table 1).

**Table 1:** Demographics, pre-migration- and post-migration data, and diagnostic categories for 55 multi traumatized outpatients with a refugee background

	**Outpatients**	
	**N**	**%**
**Background**		
Gender (male/female)	32/23	58.2/41.8
**Age groups**		
<30	7	12.7
31-45	29	52.7
>45	19	34.5
**Marital status**		
Single	10	18.2
Married	32	58.2
Divorced	13	23.6
Having children	42	76.4
**Nationality of origin**		
Eastern Europe	22	40.0
Africa	9	16.4
Middle East	14	25.5
Far East	7	12.7
Latin America	3	5.5
**Religion**		
Christian	9	16.4
Muslim	35	63.6
Other	2	3.6
No religion	9	16.4
Pre-migration*		
Number of types of trauma (M SD)	9.9	2.6
Exposure to war	42	76.4
Captivity	31	56.4
Assault with weapon	43	78.2
Physical assault	48	87.3
Severe human suffering	49	89.1
**Post-migration**		
**Years in Norway¹**		
≤10 years	9	16.7
>10 years	45	83.3
Time since trauma (M SD)	17.5	9.6
Speaking Norwegian medium-very good (self-evaluation)	49	90.7
**Education²**		
<12 years	21	38.2
12 years	9	16.4
> 12 years	23	41.8
**Employment**³		
Employed	13	25.0
Partly employed	8	15.4
Not employed	31	59.6
**Diagnostic groups**		
Affective disorders §	44	80
Anxiety disorders §§	52	94.5
Substance use disorders §§§	7	12.7
Eating disorders §§§§	3	5.5
Psychotic disorders §§§§§	1	1.8

*Note:* (*) Pre-migration variables have been selected from the LEC (Life Events Checklist) categories.

(¹) N= 54; (²) N=53; (³) N=52; (§)Any of the following diagnoses: MDD or dysthymia; (§§)Any of the following diagnoses: Panic, Agoraphobia, Social phobia, OCD, GAD or PTSD; (§§§)Any of the following conditions: Alcohol abuse disorder or Substance abuse disorder; (§§§§) Bulimia; (§§§§§) Psychosis.

### Bivariate correlations among variables of interest

As shown in Table 2, bivariate correlation analysis showed no significant relationships between PTG and demographic variables, but showed medium to strong negative correlations with posttraumatic stress and depressive symptoms, and weak social network. The correlations with unemployment and poor social integration were negative and just below the level of statistical significance.

**Table 2:** Description and bivariate correlations of study variables

**Variables**	**I**	**II**	**III**	**IV**	**V**	**VI**	**VII**	**VIII**	**IX**	**X**	**XI**	**XII**	**XIII**	**XIV**
I Age	-	-.282*	.251	.208	-.095	-.231	.212	.264	.-.238	-.127	-.135	.096	-.143	-.119
II Gender			-.082	.014	-.041	.193	-.272*	-.122	.083	.165	.077	-.162	.235	.038
III Weak social network				.450**	.192	.-468***	.346*	.500***	-.351**	-.532***	-.611***	-.558***	-.392**	-.072
IV Poor social integration^a^					.278*	-.250	.378**	.528***	-.319*	-.365**	-.477***	−396**	.-376**	−0.93
V Unemployment						-.252	.140	.229	-.343*	-.312*	-.369**	-.384**	-.194	-.111
VI PTGI-SF							-.352**	-.465***	.508***	.582***	.411**	.489***	.467***	.415**
VII IES-R								.694***	-.452**	-.529***	-.453**	-.382**	-.654***	-.341*
VIII HSCL-25-Depresion									-.587***	−583***	−542***	-.508***	-.699***	-.320*
IX Physical										.732***	.464***	.481***	.615***	.565***
X Psychological											.542***	.530***	.710***	.476***
XI Social												.621***	.387**	.070
XII Environmental													.477***	.326*
XIII Overall QoL														.672***
XIV Overall health														-
N	55	55	54	55	52	55	54	55	55	55	55	55	55	54
M	42.01	-	8.00	10.20	-	22.58	51.74	2.71	9.15	9.48	10.93	10.71	2.40	2.13
SD	9.14	-	2.91	2.56	-	10.03	17.08	0.64	2.37	2.51	3.09	2.43	0.89	0.95
Range	40	-	11	12	-	39	73	2.27	10.86	10.67	12	11	3	4

**Note**: (*) Poor social integration = Social Integration Index reversed; PTGI-SF= Posttraumatic Growth Inventory- Short Form; IES-R= Impact of Event Scale; HSCL-25-Depresion = Hopkins Symptom Checklist Scale-Depression subscale; Physical = WHO Quality of Life –Bref DOM1; Psychological = WHO Quality of Life –Bref DOM2; Social = WHO Quality of Life –Bref DOM3; Environment= WHO Quality of Life –Bref DOM4; Overall QoL = WHO Quality of Life –Bref, item 1; Overall health= WHO Quality of Life –Bref, item 2.

- Spearman’s rho nonparametric correlations were used for all variables, with the exception for the variables of gender and employment where Kendall tau b were used.

*p<0.05, **p<0.01, ***p<0.001.

Posttraumatic stress and depressive symptoms were found to be significantly and positively correlated with post-migration stress variables such as weak social network and poor social integration. The quality of life general items and domains were not correlated with any demographic variables, but were significant negatively correlated to posttraumatic stress and depressive symptoms, and all post-migration stressors, and positively correlated with PTG.

### Regression analyses for quality of life, depressive symptoms, posttraumatic stress symptoms, posttraumatic growth and unemployment

The models for all the four domains of quality of life were significant. Posttraumatic stress symptoms were not significantly associated with any of the four domains of quality of life, while gender had only one significant association with the environment domain. Depressive symptoms were significantly associated with psychological health, social relationships, and environment. Posttraumatic growth had the largest significant association with the physical health, psychological health and environment. Unemployment was significantly associated with the environment scale only. Physical health was significantly associated with posttraumatic growth only, while psychological health was significantly associated with both depressive symptoms and posttraumatic growth. Social relationships was significantly associated with depressive symptoms only, while environment was significantly associated with gender, depressive symptoms, posttraumatic growth and unemployment (see Table 3). In post-hoc power calculations, there was 86%, 92%, 17% and 48%, respectively, for the observed associations between posttraumatic growth and physical health, psychological health, social relations and environment, respectively. Thus, only the associations between posttraumatic growth and physical and psychological health had enough power to detect the observed associations. Despite the low power for the social relations and environment domains we have chosen to include these two domains in the hierarchical analysis for exploratory and descriptive purposes.

**Table 3:** Regressions predicting quality of life (WHOQOL-Bref) outcome from gender, HSCL-25-D, IES-R, PTGI-SF and unemployment

**Criterion**	**Gender**	**HSCL-25-D**	**IES-R**	**PTGI-SF**	**Unemployment**	***F***	***R²*adj.**	**95%CI**	***p***
WHOQOL-Bref DOM 1	−0.120	−0.272	−0.172	0.373**	−0.188	10.184	0.479	0.04, 16.22	p<0.001
WHOQOL-Bref DOM 2	−0.062	−0.306*	−0.190	0.392***	−0.126	12.620	0.537	9.18, 16.37	p<0.001
WHOQOL-Bref DOM 3	0.018	−0.432*	−0.031	0.140	−0.196	5.644	0.317	11.21, 21.41	p<0.001
WHOQOL-Bref DOM 4	−0.263*	−0.328*	−0.042	0.332**	−0.247*	8.604	0.432	11.28, 18.86	p<0.001

**Note**: 1) Standardized regression coefficients (Beta) for each predictor reported for the reference value for the other.

2) Unemployment has 1 degree of freedom.

3) WHOQOL-Bref DOM 1= WHOQOL-Bref physical; WHOQOL-Bref DOM 2= WHOQOL-Bref psychological.

WHOQOL-Bref DOM 3= WHOQOL-Bref social; WHOQOL-Bref DOM 4= WHOQOL-Bref environment; HSCL-25-D = Hopkins Symptom Checklist Scale-Depression subscale; IES-R= Impact of Event Scale-Revised; PTGI-SF= Posttraumatic Growth Inventory - Short Form.

*p <.05; **p<.01; ***p<.001.

N= 51.

All four domains of the quality of life questionnaire (WHOQOL-Bref) were investigated separately as dependent variables, and the independent variables were entered in four steps. On the first step in the hierarchical analysis gender accounted for 0.4% of the variance in physical health scores (F (1,49) = 0.208, p= 0.650), 1.9% for psychological health (F(1,49) = 0.924, p =0.341), 1.9% for social relationships (F (1,49) = 0.973, p = 0.329), and 1.2% for environment (F (1,49) = 0.617, p = 0.436). In step 2, depressive symptom scores and posttraumatic stress symptom scores accounted for an additional 36% of the variance in scores for physical health (F (3,47) = 9.218, p < 0.001), 41% for psychological health (F (3,47) = 12.215, p < 0.001), 30% for social relationships (F (3,47) = 7.547 p < 0.001), and 31% for environment (F (3,47) = 7.423, p < 0.001). In step 3, posttraumatic growth scores accounted for an additional 12% of the variance in scores for physical health (F (4,46) = 11.447, p < 0.001), 13% for psychological health (F (4,46) = 15.199, p < .001), 2.6% for social relationships (F (4,46) = 6.206 p < 0.001), and 6.3% for environment (F (4,46) = 8.796, p < 0.001). In the final step the unemployment variable accounted for an additional 3.2% of the variance in scores for physical health (F (5,45) = 10.184, p < 0.001), 1.5% for psychological health (F (5,45) = 12.620, p < 0.001), 3.4% for social relationships (F (5,45) = 5.644 p < 0.001), and 10% for environment (F (5,45) = 8.604, p < 0.001). All the changes were statistically significant at each step, with the exception of the first step. The final models accounted for 49% of the variance in physical health, 56% of the variance in psychological health, 34% of the variance in social relationships, and 34% of the variance in environment (See Table 4).

**Table 4:** Hierarchic regression analyses for predicting quality of life (WHOQOL-Bref)

	**WHOQOL-Bref physical**											
	**Step 1**				**Step 2**			**Step 3**			**Step 4**	
	**B**	**95%CI**	**BETA**	**B**	**95%CI**	**BETA**	**B**	**95%CI**	**BETA**	**B**	**95%CI**	**BETA**
HSCL-25-D	−2.205³	−3.07, -1.35	−0.593	−1.773²	−2.96, -0.58	−0.477	−1.18¹	−2.33, -0.03	−0.317	−0.935	−2.06, 0.19	−0.251
IES-R				−0.024	−0.07, 0.22	−0.168	−0.018	−0.06, 0.03	−0.123	−0.022	−0.06, 0.02	−0.153
PTGI-SF							0.093²	0.04, 0.15	0.395	0.085²	0.03, 0.14	0.361
Unemployment										0.651¹	0.05, 1.25	0.232
R square	0.352			0.366			0.484			0.533		
R square change	0.352			0.014³			0.118³			0.049³		
**WHOQOL-Bref psychological**												
HSCL-25-D	−2.547³	−3.41, -1.68	−0.645	−1.975²	−3.16, -0.78	−0.500	−1.321¹	−2.44, -0.20	−0.334	−1.115¹	−2.23, -0.01	−0.282
IES-R				−0.032	−0.08, 0.01	−0.209	−0.025	−0.07, 0.02	−0.162	−0.029	−0.07, 0.01	−0.186
PTGI-SF							0.102³	0.05, 0.16	0.409	0.096³	0.04, 0.15	0.383
Unemployment										0.549	−0.04, 1.14	0.184
R square	0.415			0.438			0.565			0.596		
R square change	0.415			0.023³			0.127³			0.031³		
**WHOQOL-Bref social**												
HSCL-25-D	−2.623³	−3.71, -1.53	−0.568	−2.495²	−4.02, -0.97	−0.541	−2.146²	−3.74, -0.55	−0.465	−1.856¹	−3.45, -0.27	−0.402
IES-R				−0.007	−0.07, 0.05	−0.243	−0.003	−0.06, 0.06	−0.019	−0.008	−0.07, 0.05	−0.047
PTGI-SF							0.055	−0.03, 0.13	0.187	0.045	−0.03, 0.12	0.155
Unemployment										0.771	−0.07, 1.62	0.222
R square	0.323			0.324			0.350			0.395		
R square change	0.323			0.001³			0.026³			0.045³		
**WHOQOL-Bref environmental**												
HSCL-25-D	−1.983³	−2.90, -1.07	−0.527	−2.013²	−3.30, -0.73	−0.535	−1.492¹	−2.77, -0.21	−0.397	−1.204	−2.46, 0.05	−0.320
IES-R				0.002	−0.04, 0.05	0.011	0.007	−0.04, 0.06	0.050	0.002	−0.04, 0.05	0.016
PTGI-SF							0.082¹	0.02, 0.15	0.342	0.072¹	0.01, 0.13	0.303
Unemployment										0.768¹	1,43	0.271
R square	0.278			0.278			0.367			0.433		
R square change	0.278			0.000³			0.089³			0.066³		

Note: WHOQOL-Bref physical (F(4,46) = 13.145, p<0.001; adjusted R² = 0.493); WHOQOL-Bref psychological (F(4,46) = 17.974, p<0.001; adjusted R² = 0.561); WHOQOL-Bref social (F(4,46) = 7.507, p<0.001; adjusted R² = 0.342); WHOQOL-Bref environmental (F(4,46) = 8.790, p<0.001; adjusted R² = 0.384); HSCL-25-D = Hopkins Symptom Checklist Scale-Depression subscale; IES-R= Impact of Event Scale-Revised; PTGI-SF= Posttraumatic Growth Inventory - Short Form.

¹p <.05; ²p<.01; ³p<.001.

## Discussion

### Growth and psychopathology

In this clinical study of multi-traumatized refugees we found both salutogenic and pathological outcomes as a result of exposure to multiple traumatic events. All patients reported some degree of posttraumatic growth, but only near third of them reported great degrees of change, while the majority of the patients reporting PTSD symptoms and depressive symptoms at clinically significant levels. The posttraumatic growth we observed was lower than has been observed among Somali refugees in Hungary [10], Latino immigrants in USA [121], Tibetan refugees in India [11], multiracial veterans from the Iraq war [20], and survivors of a disaster on an oil platform [39], but slightly higher than among Bosnian refugees in Sarajevo [9]. The level of growth found among our patients may be explained by the high number of exposures to traumatic events, and the long length of time since the traumatic exposures. PTG takes time to develop [5,122,123] and is a process that may imply several developmental stages in order to reach an authentic growth, such that with the passing of time an increase in growth could be expected, as has been previously reported in a study of out-patients with head injuries [124].

The level of posttraumatic stress symptoms was higher than has been identified among war refugees from the former Yugoslavia resettled in the West [125], and among an adult population from Kabul, Afghanistan [126]. On the other hand, these levels were lower than what has been found among Bosnian refugees [127] and refugees with PTSD from several countries resettled in Norway [128]. We found a significant, medium negative correlation between PTG and PTSD symptoms in our patients who were exposed to traumatic events many years ago, suggesting that the passage of time aids in the development of an authentic growth that should be negatively related to psychopathology [16,129]. In a meta analysis Helgeson observed that “relationships between PTG and psychological distress suggest that over time reports of growth are related to better psychological health and by implication are more likely to reflect authentic growth” ([31] page 74).

We believe action mediated growth theory [16] offers our population of refugees the best framework for understanding our results. The majority of our patients were exposed to traumatic events several years ago in their home countries, and because of these traumas many of them chose to immigrate to Norway to find a better and safer place to live. Thus, migration can be seen as an act of conscious decision-making and as an action of growth based on the developing cognition to change one’s life for the better. Furthermore, the extended length of time since the traumatic exposures in our patients implies a stabilization of the process of posttraumatic growth, which other studies have reported to be stable over longer periods of time [44,130].

Several studies have also found a negative correlation between growth and PTSD symptoms in populations exposed to war [15,42,131], in victims of interpersonal violence [27,132], and in victims of terrorist attacks [18,133].

With the passing of time after a traumatic event there is also an increased risk for the development of depressive symptoms, and several studies have found a negative association between posttraumatic growth and depression [52,134,135]. We found a negative medium-to-large negative correlation between PTG and depressive symptoms, and may thus conclude some show of support for our first hypothesis that PTG is negatively associated with psychopathological symptoms.

### Growth, psychopathology and quality of life

All the domains of quality of life had below average scores, with the lowest scores on the “Overall health” general item and the highest for the “Social relations” domain. Our findings for the levels of quality of life were lower than the levels found among Iranian refugees resettled in Sweden [66], among Turkish immigrants in Sweden [136], Haitian immigrants in USA [137], traumatized refugees resettled in the Netherlands [138,139],and Turkish immigrants in London [140], but lower than in tortured refugees resettled in Denmark [64,65,141]. Following the first hypothesis, we found moderate-to-large, positive and significant correlations between PTG and all of the domains of quality of life, including the two general items of “Overall quality of life” and “Overall health”. Several studies have also found positive correlations between PTG and quality of life among ethnic minorities [31], cancer survivors [50,51,53], HIV patients [142], and traumatized students [54]. To date there are few studies among refugee populations where both PTG and quality of life have been assessed. We therefore must compare our findings with studies conducted among other traumatized populations and take into account the likely differences due to exposure to different kinds of traumas relative to those seen in our population.

The second hypothesis proposed negative correlations between psychopathological symptoms and quality of life, as well as positive correlations with post-migration stressors. Again, in accordance with this hypothesis we found medium-to-large significant negative correlations between all quality of life domains and posttraumatic stress and depressive symptoms. This finding is in line with previous studies that found the same pattern of negative correlations between quality of life or well being and psychopathological symptoms among war-wounded refugees [62] and war veterans [59,143]. Further, we observed medium-to-large significant positive correlations between psychopathological symptoms and post-migration stressors, like weak social network and poor social integration. A Norwegian study among a refugee population found a negative correlation between negative symptoms and both posttraumatic stress and depressive symptoms, as well as social network [71]. Acculturation stress has been found to be positively correlated with negative mental health symptoms in several refugee and immigrant studies [91-94,144]. A study among resettled Sudanese refugees in Australia found a negative correlation between posttraumatic stress symptoms and both social support and employment [70]. Unemployment was positively correlated with both posttraumatic stress and depression symptoms, but the correlations were not statistically significant. Several other refugee studies found positive correlations between psychopathological symptoms and unemployment [84-87]. Further, we found that unemployment was negatively significant correlated with all the domains from the quality of life. Many studies have investigated the relationship between unemployment and quality of life and have found lower levels of physical and psychological well-being among unemployed participants compared to employed participants [79]. Unemployment is therefore an important issue to address in refugees resettled in a Western country, and it should be a priority for both therapists working with this patient group and for local authorities in order to help improve the integration process and the quality of life of these refugees. Even though unemployment was not statistically significantly correlated with psychopathological symptoms in our sample, we may conclude based on previous findings that our second hypothesis has some support.

We aimed to assess the associations between quality of life, posttraumatic growth and posttraumatic stress symptoms. All quality of life indicators were negatively associated with posttraumatic stress symptoms and depression symptoms, while posttraumatic growth was positively associated with all of the quality of life indicators. The association between posttraumatic growth and three of the domains of quality of life (physical, psychological and environmental) was stronger than the psychopathological symptoms of depression and posttraumatic stress. To our knowledge this is the first study which has assessed the concerted associations of both positive and negative changes with quality of life among refugees. This finding points to the important relationship between positive changes and quality of life, thus raising the issue of the value of using positive psychology and a focus on growth during therapy with multi-traumatized refugees [145-147] alongside the more traditional trauma therapies.

### Growth, psychopathology, quality of life and post-migration stressors

Most studies of quality of life in refugees have focused mainly on the associations between quality of life and posttraumatic stress and depression. Relatively few studies have assessed the association of post-migration stressors with quality of life. Consistent with other studies, we found significant negative correlations between post-migration stressors and quality of life [64,66,141,148,149]. Further, few studies among refugee populations have investigated the association between post-migration stressors and posttraumatic growth, but evidence is accumulating that stressors in the resettlement process may not only give rise to posttraumatic stress and depressive symptoms, but also to positive changes and growth [147].

We also observed a significant negative correlation between poor social integration and unemployment, and a positive correlation between posttraumatic growth and weak social network. Our findings are in agreement with other studies which found positive correlations between growth and social support among traumatized Somali refugees [10] and former prisoners of war [25].

The above results suggest support for our third hypothesis, even if the correlations between posttraumatic growth and two of the post-migration stressors were not statistically significant.

### A model for quality of life

The second aim of the study was to test a model for the quality of life among multi-traumatized refugees taking into account the total effect of demographics, symptom load, positive changes and post-migration stress factors. These variables were chosen based on theoretical considerations and because of the limited previous research showing the importance of each on quality of life. In a set of multiple regression analyses we identified significant associations between depressive symptoms, posttraumatic growth, unemployment and the quality of life scores. Posttraumatic stress symptoms had no significant association with quality of life, while gender and unemployment had only one significant association with quality of life. Posttraumatic growth had the strongest association with several of the domains of quality of life, while posttraumatic stress symptoms had the least. This finding is surprising given the broad range of therapies used with multi-traumatized refugees that are intended to reduce posttraumatic stress symptoms but do not address positive changes after the trauma. If our finding can be replicated in other studies this may give therapists working from a positive psychological perspective support for the value of their approach in treating multi-traumatized refugees. This finding again points to the necessity of addressing specific posttraumatic growth issues in therapy with multi-traumatized refugees.

To investigate the individual associations of each of the variables with the domains of quality of life, we fitted four hierarchical regressions. We found posttraumatic growth to have the strongest association with physical health, psychological health and environment, while depressive symptoms were strongly associated with psychological health and social relationships. The models explained a variance in the quality of life domains between 34% for the social relationships and 56% for psychological health. It is possible that an even larger variance in the psychological health domain could have been observed if we had taken into account the influences of more demographic variables such as age or education, or pre-migration variables such as number of exposures to traumatic events and duration of traumas, as well as post-migration variables such as years of residency in Norway and level of income. The smaller variance found in the social domain could be due to tautological effects between this domain and the social integration index used in the model.

### Methodological limitations

It is important to acknowledge some limitations in the present study. The sample size was small and any generalizability of the findings must be done with caution. Also, the cross-sectional nature of this study does not allow for causal interpretations. Our measure of PTG is an abbreviated measure of PTG, the PTGI-SF, which [111,150] may be limited in measuring growth in our population. Another measurement limitation is the reliance upon only one item to measure social network size, which likely does not catch all the aspects of one’s social network. The retrospective nature of the PTG measurement has been criticised for potentially including errors due to self-reported bias, cognitive biases, recollection distortions due to the passing of time and inaccurate measurement of changes from pre- to post-trauma [151]. In our study, the traumas had occurred many years before the measurement of posttraumatic growth, which inevitably may have contributed to recollection problems. Because of the limited sample size, we studied only a linear relationship between posttraumatic growth and posttraumatic stress symptoms, but there is also the possibility of a curvilinear relationship as has been found in several other studies. This limitation may have distorted our findings. The cross-cultural validity of the PTGI-SF has been investigated and shown to be satisfactory, however, due to the individualistic culture in which this concept was operationalized, this measurement may not be equally applicable to a collectivistic culture, which many of our patients came from [152]. Lastly, the low Cronbach’s alpha coefficient of the social domain of the WHOQOL-Bref limits the reliability of the findings for this domain.

## Conclusion

The majority of clinicians working with traumatized refugees have as their primary focus the reduction of symptoms in their patients, but very few are working to assess the positive changes after trauma and to monitor the quality of life among their patients. Positive psychological interventions among multi-traumatized patients may be successfully integrated along with therapeutic work towards psychopathological symptoms reduction, thus making use of individual strengths to improve treatment outcomes and increase the patient’s quality of life.

## Competing interests

The authors declare that they have no competing interests.

## Authors contributions

D-ST conducted the clinical interviews, performed the statistical analysis together with TW-L, and drafted the manuscript. EH, TH, LL, JS, conceived the study and its design and contributed to the manuscript. All the authors have read and approved the final manuscript.
